# Pathologic stratification of operable lung adenocarcinoma using radiomics features extracted from dual energy CT images

**DOI:** 10.18632/oncotarget.13476

**Published:** 2016-11-21

**Authors:** Jung Min Bae, Ji Yun Jeong, Ho Yun Lee, Insuk Sohn, Hye Seung Kim, Ji Ye Son, O Jung Kwon, Joon Young Choi, Kyung Soo Lee, Young Mog Shim

**Affiliations:** ^1^ Department of Radiology and Center for Imaging Science, Samsung Medical Center, Sungkyunkwan University School of Medicine, Seoul 135-710, Korea; ^2^ Department of Pathology, Kyungpook National University Medical Center, Kyungpook National University School of Medicine, Daegu 702-210, Korea; ^3^ Biostatistics and Clinical Epidemiology Center, Samsung Medical Center, Sungkyunkwan University School of Medicine, Seoul 135-710, Korea; ^4^ Division of Respiratory and Critical Medicine of the Department of Internal Medicine, Samsung Medical Center, Sungkyunkwan University School of Medicine, Seoul 135-710, Korea; ^5^ Department of Nuclear Medicine, Samsung Medical Center, Sungkyunkwan University School of Medicine, Seoul 135-710, Korea; ^6^ Department of Thoracic and Cardiovascular Surgery, Samsung Medical Center, Sungkyunkwan University School of Medicine, Gangnam-gu, Seoul 135-710, Korea

**Keywords:** lung adenocarcinoma, heterogeneity, radiomics, texture analysis, dual energy CT

## Abstract

**Purpose:**

To evaluate the usefulness of surrogate biomarkers as predictors of histopathologic tumor grade and aggressiveness using radiomics data from dual-energy computed tomography (DECT), with the ultimate goal of accomplishing stratification of early-stage lung adenocarcinoma for optimal treatment.

**Results:**

Pathologic grade was divided into grades 1, 2, and 3. Multinomial logistic regression analysis revealed *i*-uniformity and 97.5th percentile CT attenuation value as independent significant factors to stratify grade 2 or 3 from grade 1. The AUC value calculated from leave-one-out cross-validation procedure for discriminating grades 1, 2, and 3 was 0.9307 (95% CI: 0.8514–1), 0.8610 (95% CI: 0.7547–0.9672), and 0.8394 (95% CI: 0.7045–0.9743), respectively.

**Materials and Methods:**

A total of 80 patients with 91 clinically and radiologically suspected stage I or II lung adenocarcinoma were prospectively enrolled. All patients underwent DECT and F-18-fluorodeoxyglucose (FDG) positron emission tomography (PET)/CT, followed by surgery. Quantitative CT and PET imaging characteristics were evaluated using a radiomics approach. Significant features for a tumor aggressiveness prediction model were extracted and used to calculate diagnostic performance for predicting all pathologic grades.

**Conclusions:**

Quantitative radiomics values from DECT imaging metrics can help predict pathologic aggressiveness of lung adenocarcinoma.

## INTRODUCTION

Non-small cell lung cancer (NSCLC) accounts for 85% of lung cancers and adenocarcinoma is the predominant histologic subtype of lung cancer. Early-stage lung adenocarcinoma shows broad spectrum of prognosis, and moreover, reported low survival rates in a substantial proportion of patients [1–3]. Reflecting the histologic heterogeneity of lung adenocarcinoma, there is an increasing body of evidence that sublobar resection may achieve oncologic outcomes similar to those of lobectomy in early-stage NSCLC [4], although some studies have reported the contradictory result that postoperative adjuvant chemotherapy improves the prognosis even in operable early-stage NSCLC [5].

Radiomics is an ongoing field of research that enables us to obtain additional information from standard medical images using computational post-processing techniques. In an effort to enable more accurate molecular and genetic profiling of tumors, many studies have been performed with the aim of predicting treatment response and, furthermore, accomplish a step toward personalized medicine. Al-Kadi et al. showed that computed tomography (CT) features with texture analysis can be helpful in differentiating aggressive from nonaggressive NSCLC [6], and Kido et al. showed differences between histologic subtypes of peripheral bronchogenic carcinoma using textural parameters on CT [7]. Accordingly, we might expect radiomics to provide noninvasive analysis of lung adenocarcinoma and allow more effective evaluation of tumor aggressiveness based on tumor grade.

Therefore, the purpose of our study was to evaluate the usefulness of surrogate biomarkers as predictors of histopathologic tumor grade with radiomics data obtained from dual energy CT (DECT), with the ultimate aim of patient stratification for optimal treatment.

## RESULTS

### Correlation between pathologic stage and other pathologic features

Among stage 1A tumors, 6/72 (8%) were grade 3 with mainly micropapillary or solid subtype. The extent of invasion showed a positive correlation with pathologic stage (*P* < 0.01). In particular, the extent of invasion in stage 1A was 12.9 ± 7.6, which was significantly lower than that of stage 1B, 2A, and 2B. Tumor cellularity in all pathologic stages was 46.7 to 60.0 without significant difference among stages 1A through 2B. The relationships between pathologic stage and pathologic features are shown in the Supplementary Table S1.

### Correlation between imaging parameters and pathology

Table 1 shows comparisons of all CT and PET parameters according to three pathologic tumor grades. A significant difference in various parameters including SUVmax, size, and density of the nodule was found among tumors of different pathologic grade (all *P* < 0.01). A multinomial logistic regression analysis with the stepwise variable selection procedure was performed for significant factors according to the univariate analysis, and two CT factors, *i*-uniformity and 97.5th percentile CT attenuation value, were shown to be independent significant factors to stratify the three grades. On multivariate analysis, there was no variable showing multicollinearity (VIF. 10) on VIF analysis; thus, no variable was removed from the multivariate analysis for that reason. As shown in Table 2, *i*-uniformity was a significant factor in stratifying grade 2 from grade 1 (OR = 0.037 and *P* < 0.01), and 97.5th percentile CT attenuation value was a significant factor in stratifying grade 2 from grade 1 (OR = 1.006 and *P* < 0.01) and grade 3 from grade 1 (OR = 1.041 and *P* = 0.02).

**Table 1:** Characteristics of early-stage lung adenocarcinoma according to histologic tumor grade

Variables	Grade 1 (*n* = 19)	Grade 2 (*n* = 65)	Grade 3 (*n* = 7)	*P*
Age (y)	57.8 ± 8.9	58.9 ± 9.0	57.6 ± 4.2	0.82
Male:female ratio**	12 : 7	26 : 39	6 : 1	0.49
Smoking habits				0.93
Ever/Never	10/9	19/46	6/1	
PET parameter				
SUVmax	0.44 ± 0.46	3.1 ± 2.67	5.44 ± 3.84	< 0.01^*^
CT parameters				
Solidity				< 0.01^*^
Non-solid (*n* = 39)	16	23	0	
Part-solid (*n* = 22)	1	19	2	
Solid (*n* = 30)	2	23	5	
Physical Factors				
Size in lung setting (mm)	15.58 ± 7.76	25.15 ± 11.59	22.57 ± 9.54	0.28
Size in mediastinal setting (mm)	2.68 ± 6.90	13.57 ± 13.11	19.86 ± 11.19	< 0.01^*^
Volume (cm^3^)	2.20 ± 2.91	6.88 ± 10.28	7.14 ± 9.80	0.76
Density	0.41 ± 0.17	0.72 ± 0.21	0.82 ± 0.21	< 0.01^*^
*i*-Density	1.17 ± 0.02	1.03 ± 0.39	0.83 ± 0.55	< 0.01^*^
*g*-Density	1.88 ± 0.17	1.54 ± 0.22	1.43 ± 0.20	< 0.01^*^
Mass (g)	0.83 ± 1.12	5.23 ± 7.37	6.31 ± 8.64	0.79
*i*-Mass (g)	2.10 ± 2.58	5.38 ± 7.90	7.07 ± 10.77	0.29
*g*-Mass	3.79 ± 5.53	9.39 ± 17.20	10.22 ± 13.24	0.37
Histogram analysis				
Skewness	0.41 ± 0.66	−0.67 ± 1.01	−1.17 ± 0.48	< 0.01^*^
*i*-Skewness	1.19 ± 1.48	0.29 ± 0.71	−0.20 ± 0.40	0.02^*^
*g*-Skewness	−0.31 ± 0.60	0.52 ± 0.79	0.98 ± 0.83	< 0.01^*^
Kurtosis	3.37 ± 1.83	4.04 ± 4.43	3.36 ± 2.05	0.02^*^
*i*-Kurtosis	8.17 ± 8.36	4.13 ± 2.88	2.88 ± 1.70	< 0.01^*^
*g*-Kurtosis	3.15 ± 1.71	3.77 ± 2.60	3.85 ± 1.83	< 0.01^*^
75th percentile (HU)	−496 ± 192	−175 ± 210	43.3 ± 133	< 0.01^*^
*i*-75th percentile (HU)	66.1 ± 25.1	75.7 ± 33.7	50.0 ± 27.1	< 0.01^*^
*g*-75th percentile	734 ± 119	471 ± 205	379 ± 218	< 0.01^*^
97.5th percentile (HU)	−329 ± 171	−49.3 ± 151	67.8 ± 45.5	0.01^*^
*i*-97.5th percentile (HU)	135.7 ± 25.2	134 ± 58.5	76.0 ± 61.5	< 0.01^*^
*g*-97.5th percentile	838 ± 55.9	687 ± 182	780 ± 103	< 0.01^*^
Texture analysis				
Uniformity	0.0046 ± 0.0034	0.0028 ± 0.0019	0.0020 ± 0.0031	0.08
*i*-Uniformity	0.0134 ± 0.0046	0.0071 ± 0.0047	0.0061 ± 0.0046	< 0.01^*^
*g*-Uniformity	0.0057 ± 0.0039	0.0031 ± 0.0017	0.0031 ± 0.0018	0.48
Entropy	8.26 ± 0.91	8.85 ± 1.26	7.99 ± 2.71	0.10
*i*-Entropy	6.64 ± 0.49	6.38 ± 2.39	5.13 ± 3.42	< 0.01^*^
*g*-Entropy	7.95 ± 0.99	8.78 ± 0.68	8.86 ± 0.83	0.40
Gradient-variability				
*g*-Intensity-variability	7.42 ± 3.84	7.97 ± 4.28	9.00 ± 2.41	0.08
*g*-Size-zone-variability	8.97 ± 8.18	15.7 ± 12.5	18.4 ± 13.9	0.59

Note. Classified according to the International Multidisciplinary Lung Adenocarcinoma Classification system.

Unless otherwise indicated, data are means ± standard deviation.

* *P* < 0.05.

**Table 2:** Multinomial logistic regression analysis for stratifying three pathologic tumor grades

Imaging variable	OR (95% CI)	*P* value
*i-*Uniformity		
Grade 1	1	
Grade 2	0.037 (0.003–0.419)	< 0.01^**^
Grade 3	0.110 (0.006–1.887)	0.13
97.5th percentile (HU)		
Grade 1	1	
Grade 2	1.006 (1.002–1.010)	< 0.01^**^
Grade 3	1.041 (1.006–1.077)	0.02^*^

Note. *CI* = confidence interval.

* *P* < 0.05.

** *P* < 0.01.

### Predictive probability of quantitative CT parameters for stratifying tumor grades

**Figure 1:**
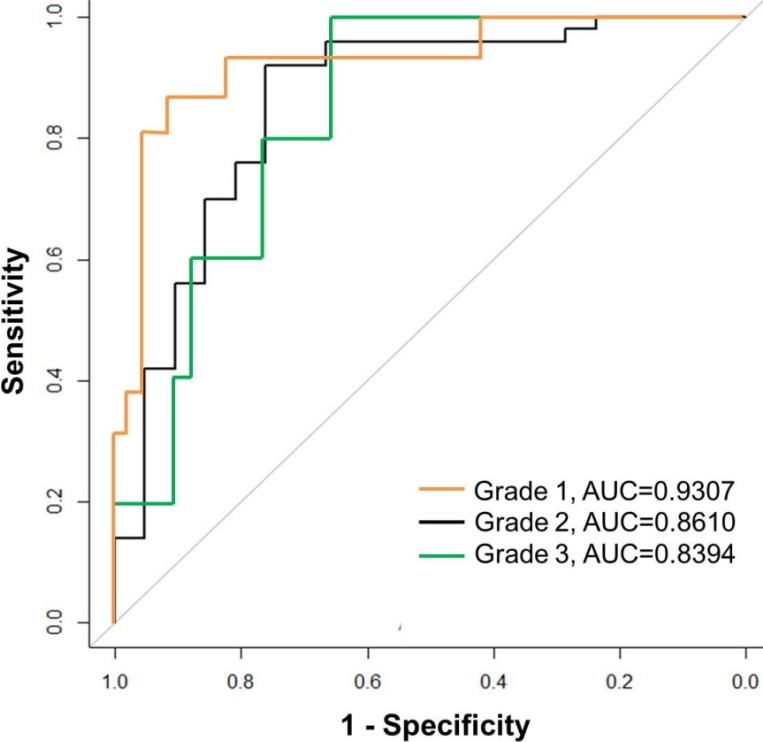
Receiver operating characteristic (ROC) curves for prediction of pathologic grade based on significant imaging parameters The AUC calculated from leave-one-out CV for discriminating grade 1 from the other grades was 0.9307, which was the highest among three ROC curves. The AUC was 0.8610 for discriminating grade 2 and 0.8394 for discriminating grade 3.

Finally, we used leave-one-out CV procedure to evaluate the accuracy of prediction of pathologic grade and we constructed ROC curves and calculated AUC. (Table 3 and Figure 1). The AUC value calculated from leave-one-out CV for discriminating grade 1 from the other grades was 0.9307 (95% CI: 0.8514–1), which was the highest among the three ROC curves. The AUC for discriminating grade 2 from the other grades was 0.8610 (95% CI: 0.7547–0.9672) and the AUC for discriminating grade 3 was 0.8394 (95% CI: 0.7045–0.9743).

**Table 3:** Predictive probability of quantitative CT parameters for stratifying tumor grades

Pathologic grade	Sensitivity (%)	Specificity (%)	PPV (%)	NPV (%)
Grade 1	75.0 (0.500–0.938)^*^	96.3 (0.909–1.000)^*^	85.7 (0.688–1.000)^*^	93.0 (0.871–0.982)^*^
Grade 2	96.0 (0.900–1.000)^*^	57.1 (0.381–0.762)^*^	84.2 (0.783–0.909)^*^	85.7 (0.688–1.000)^*^
Grade 3	0	100 (1.000–1.000)^*^	0	93.0 (0.930–0.930)^*^

Note. PPV = positive prediction value, NPV = negative prediction value.

* Data in parentheses are the confidence interval.

## DISCUSSION

Radiomics is an emerging field that converts medical imaging data of a tumor to quantitative biomarkers by the application of advanced computational methodologies [8, 9]. Quantitative imaging features that are extracted from the defined tumor region include descriptors of intensity distribution, spatial relationships between the various intensity levels, and texture heterogeneity patterns [10]. For example, Win et al. [11] reported that CT-derived tumor textural heterogeneity and PET-derived textural heterogeneity were independent predictors of survival. In a radiomics analysis of 440 features extracted from CT data of patients with lung or head-and-neck cancer, [8] a large number of radiomics features were proven to have prognostic power. The data of this study by Aertz et al. [8] suggested that by capturing intratumoral heterogeneity, which is associated with underlying gene expression patterns, radiomics identifies a general prognostic phenotype existing in both lung and head-and-neck cancer. Therefore, radiomics features are expected to have the potential to capture intratumoral heterogeneity and, furthermore, the distinct phenotypic differences of tumors, and have been proven to have prognostic power with clinical significance [8, 10–13].

The prognostic impact of pathologic invasiveness and the need to predict pathologic invasiveness have been described in many studies. Histologic subtypes defined by IASLC/ATS/ERS classification and other pathologic factors such as lymph node involvement and pleural and vessel invasion are known to show correlation with the survival outcome [14, 15]. Consequently, in the clinical setting accurate prediction of pathologic invasiveness may aid the decision for an appropriate operation type based on the status of the tumor. Major lung resection has been recommended as a standard procedure for the treatment of lung adenocarcinoma, but according to recent literature limited surgical resection could be used instead for cases of small-sized lung cancer [16–20]. Meanwhile, some studies have emphasized the importance of stratifying early-stage NSCLC patients to select candidates for adjuvant therapy [14, 15]. The presence of the micropapillary or solid subtype has been shown to be a poor prognostic factor for overall survival and for recurrence in patients with NSCLC [21, 22] and Travis et al. [21] suggested that micropapillary or solid predominant subtype predicts improved responsiveness to adjuvant chemotherapy compared with acinar or papillary predominant tumors in patients with surgically resected lung adenocarcinoma [23].

A major problem with assessing pathologic invasiveness and grade in lung adenocarcinomas has been that these features can be estimated using a whole tumor specimen from complete resection, but not using core biopsy or cytologic material [24–26]. Of course, there have been efforts to provide prognostic information using limited biopsy samples from lung cancer patients, but biopsy samples frequently contain limited amounts of cancer tissue. In fact, according to Coghlin et al., the mean percentage area of tumor in a biopsy sample was only 33.4% [27]. More importantly, our results indicated that tumor cellularity represented only 46.7 to 60.0% of the whole tumor volume. Moreover, the tumor area is not homogenous but tumors themselves are spatially and temporally heterogeneous, therefore complete evaluation of the tumor status is not easily achieved. In this situation radiomics, which can provide a comprehensive view of the entire tumor without invasive procedures, could be the key to achieve full evaluation of tumor status including tumor aggressiveness and also its prognostic power.

However, radiomics still has barriers to its implementation in clinical practice. According to Nyflot et al. [28], various imaging parameters such as acquisition noise, phantom size and reconstruction method show substantial influence on textural features. In addition, since the manual nodule segmentation is yet a gold standard, it suffers from significant inter-reader bias and low reproducibility [29]. To overcome the limitation of radiomics, variation of image acquisition, segmentation and analysis procedures should be minimized.

We aimed to determine the potential of radiomics data from DECT imaging metrics for tumor stratification compared with pathologic results from completely resected tumors. The DECT technique allows differentiation of an iodine substance from other materials by the material decomposition principle [30]. The real iodine component of the tumor can be measured on iodine-enhanced images of DECT and is comparable to the real value of the extent of enhancement [31]. Tumor angiogenesis, which leads to increased microvessel density, results in increased tumor perfusion and thus iodine enhancement [32, 33]. Therefore, the net iodine value obtained through DECT may reflect the level of underlying tumor angiogenesis [34]. On the other hand, a recent study demonstrated that the iodine-related attenuation of DECT in primary lung cancer correlates with the SUVmax of PET/CT because increased glucose metabolism, which is known to have an association with tumor perfusion, increases FDG uptake in PET [32]. These authors also showed that the iodine value could be added to conventional FDG PET/CT for more advanced tumor grading [32]. Overall, with radiomics values derived from DECT and PET/CT, tumor phenotyping with consideration of the tumor metabolism and perfusion could be established in our study.

Our study had several limitations. First, this study was performed at a single institution and therefore the patient population was relatively small. Especially, grade 3 group included only 7 tumors, which is very small and therefore it may have affected the reliability of our results. Further studies with large numbers of patients from multiple centers are needed to confirm the feasibility of DECT and its quantitative analysis for prediction of tumor grade and aggressiveness. Second, the pathologic tumor grades 1, 2, and 3 used in this study only reflect the most predominant subtype of the tumor, without consideration of the presence of other subtypes within the tumor. Future studies should be performed with consideration of tumor heterogeneity and the proportion of the histologic subtype, which can affect the prognosis. Third, potential disagreement between the pathologists in pathologic evaluation was present in this study. However, effort was made to minimize the disagreement since the pathologic assessment was the standard of reference in our study. Two pathologists reviewed the cases independently, followed by reaching consensus in a two-step order. In addition, digital miscroscope system was used to obtain objectivity of visual assessment.

In conclusion, quantification using preoperative DECT imaging metrics can help to predict pathologic aggressiveness based on tumor grade. Among various CT radiomics parameters obtained from DECT, *i*-uniformity and 97.5th percentile CT attenuation value were proved to be independent significant factors for predicting tumor grade by distinguishing grade 2 or 3 from grade 1.

## MATERIALS AND METHODS

### Patients

This study was performed as part of an ongoing prospective clinical trial that aimed to determine the value of biomarkers for the prognostic stratification in patients with early-stage lung adenocarcinoma at a single tertiary referral hospital (NCT01482585). This prospective study was approved by the institutional review board and written informed consent was obtained (No. SMC 2011-09-083).

Between October 2011 and May 2013, a total of 101 patients with stage I or II lung adenocarcinoma were prospectively enrolled. Inclusion criteria for our study were as follows: (1) Clinically and radiologically suspected lung adenocarcinoma, (2) Newly-diagnosed stage I or II disease from clinical work-up including F-18-fluorodeoxyglucose (FDG) positron emission tomography (PET)/CT, (3) Eastern Cooperative Oncology Group (ECOG) performance status of 0 or 1 who are eligible for surgery, (4) Age 20 years or older, (5) Able to tolerate DECT imaging as required per protocol, (6) Able to give study-specific informed consent. Exclusion criteria were (1) Prior malignancy, (2) Scheduled for definitive radiation therapy or neoadjuvant concurrent chemoradiation therapy, (3) Poor cardiopulmonary reserve (contraindication for surgery).

**Figure 2:**
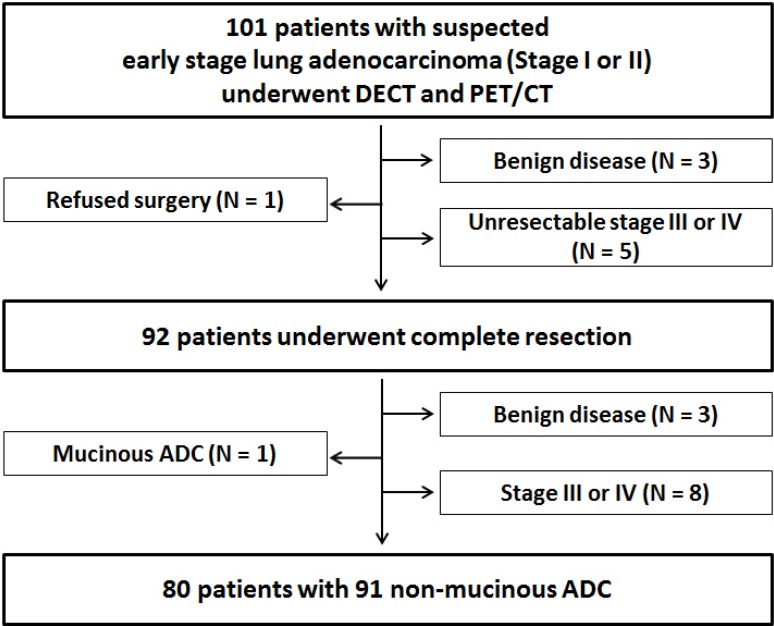
Flow chart of the study population ADC = adenocarcinoma.

All 101 consecutive patients underwent DECT and FDG PET/CT for work-up. Among 101 enrolled patients, 3 patients who were proven to have benign disease after percutaneous biopsy of the lesion and 5 patients who were proven to have unresectable stage III or stage IV lung cancer through further studies were excluded. In addition, one patient who refused surgery was excluded. Overall, 92 patients underwent complete resection and 3 patients with benign disease, 8 patients with pathologic stage III or IV disease, and 1 patient with mucinous adenocarcinoma were additionally excluded. Further details are described in the Supplement. Finally, 80 patients with 91 stage I or II lung adenocarcinomas were included in our analysis (Figure 2). The clinicopathologic characteristics of the 80 patients and 91 lung adenocarcinomas included in this study are summarized in Table 4.

**Table 4:** Clinicopathologic characteristics of early-stage lung adenocarcinoma (91 tumors of 80 patients)

Characteristics	Number of patients/nodules
Gender (%)	
Male	37 (46)
Female	43 (54)
Median age (y)	58 (35–76)^*^
Smoking habits (%)	
Non-smoker	50 (63)
Current/former smoker	30 (37)
Type of operation^†^	
Segmentectomy	27 (30)
Lobectomy	64 (70)
p-T status^†^	
≤ 2 cm	53 (58)
>2 cm	38 (42)
p-N status	
N0	87 (96)
N1	4 (4)
Histopathology^†^	
AIS	4 (5)
MIA	11 (12)
Invasive adenocarcinoma	76 (83)
Lepidic predominant	5 (7)
Acinar/Papillary predominant	64 (84)
Micropapillary/Solid predominant	7 (9)
Pathologic stage	
1A	72 (58)
1B	15 (21)
2A	3 (19)
2B	1 (2)

Note. Unless otherwise indicated, data in parentheses are percentages.

AIS = adenocarcinoma *in situ*, MIA = minimally invasive adenocarcinoma.

* Data in parentheses are the range.

† Data are numbers of tumors (*n* = 91).

### Imaging and Analysis

**Figure 3:**
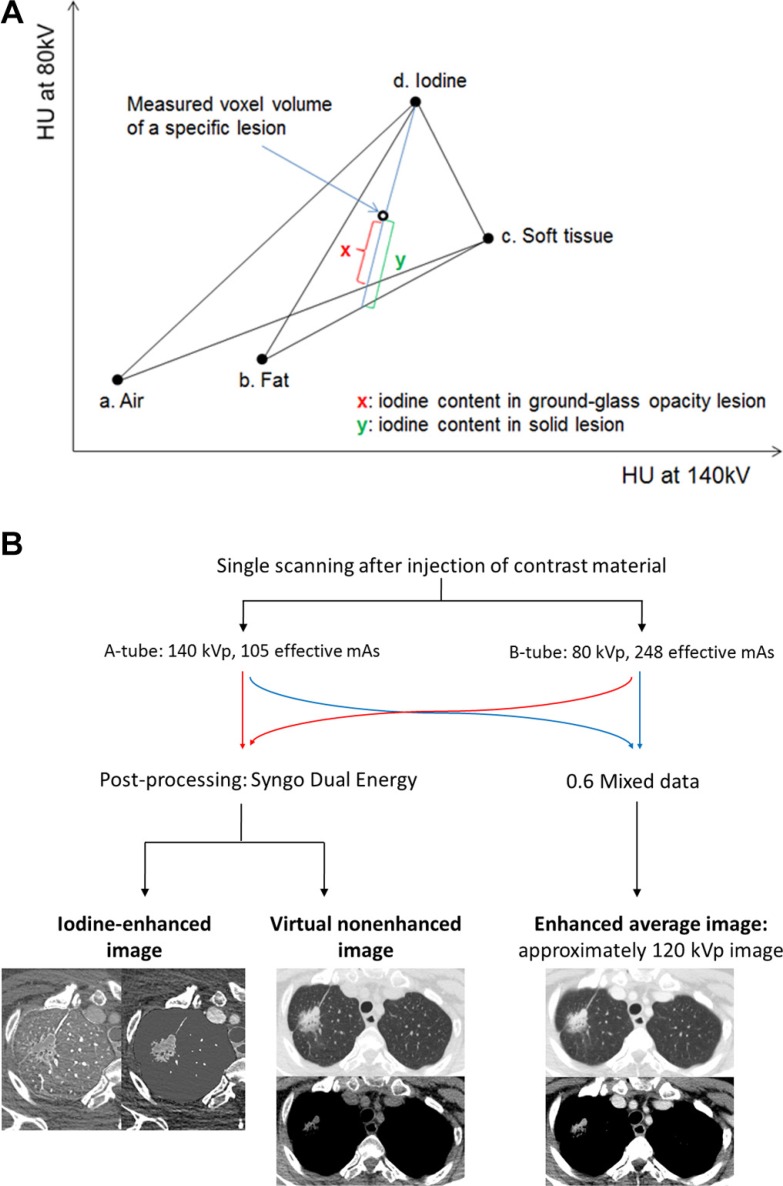
Overview of dual-energy imaging (**A**) Diagram of three-material decomposition of voxel used by dual-energy software. (a), (b), (c), and (d) are fixed points of CT attenuation values from two different energies for air, fat, soft tissue, and iodine. Intercept x or y along iodine axis represents iodine content of voxel on this two-energy plot. (x) is the degree of enhancement of ground glass opacity nodule, whereas (y) is the degree of enhancement of solid nodule. (**B**) Three types of data sets were generated from the DECT scanning: the 80 kV, 140 kV, and enhanced weighted-average images. The weighted-average images were generated by combining the 140-kV and 80-kV data sets with a weighting factor of 0.6 (60% of the information derived from the 80 kV image and 40% derived from the 140 kV image) and these were approximately 120 kV images. The virtual non-enhanced images and iodine-enhanced images were made by using the liver Virtual Non- Contrast (VNC) application mode of dedicated dual-energy postprocessing software (Syngo Dual Energy; Siemens Medical Solutions, Forchheim, Germany).

All patients underwent CT examination using a dual-source CT scanner (Somatom Definition Flash; Siemens Healthcare, Forchheim, Germany) with the dual-energy technique. The overview of dual-energy imaging is described in Figure 3. Three types of data set were generated from the DECT scanning: 80 kV, 140 kV, and enhanced weighted-average images. Further details of image parameters are described in the Supplement. PET/CT images were acquired using a Discovery STe scanner (GE Healthcare, Milwaukee, WI). Unenhanced CT was performed with 16-slice helical CT (140 keV, section width of 3.75 mm, 30–170 mA in AutomA mode) at 1 h after injection of 18F-FDG (5.0 MBq/kg) and emission scan was performed at 2.5 min per frame in 3D mode. PET images were reconstructed using a 3D ordered subsets expectation-maximization algorithm (voxel size, 3.9 × 3.9 × 3.3 mm3). The standardized uptake value (SUV) was calculated by correcting for the injected dose of 18F-FDG and body weight. Virtual non-enhanced images and iodine-enhanced images were generated using the liver Virtual Non-Contrast (VNC) application mode of dedicated dual-energy post-processing software (Syngo Dual Energy; Siemens Medical Solutions, Forchheim, Germany). To obtain the iodine value of both the solid and ground-glass opacity (GGO) component in each tumor, postprocessing was performed with two different types of software. Image data were reconstructed with a section thickness of 1 mm using a D30f (medium smooth) kernel for the iodine-enhanced image and a D45f (medium sharp) kernel for the virtual non-enhanced image.

CT scans were assessed for the type of nodule in terms of GGO nodule (GGN), part-solid nodule, or solid nodule. Nodule size in both the lung setting and mediastinal setting was evaluated manually.

The stability of various quantitative CT features with intra-observer reliability was evaluated through calculation of intra-class correlation coefficients (ICC) in 25 randomly selected patients [35]. The concordance correlation coefficient (CCC) was calculated for each feature using this test-retest image set. Features whose CCC is less than 0.8 were not considered reproducible and were excluded from our analysis. For nodule segmentation, tumors were segmented independently by two chest radiologists who were unaware of clinical and pathologic results by drawing a region of interest (ROI) covering as large an area of the whole tumor as possible. Quantitative CT analysis was performed based on physical, histogram-based, regional, and local features from the manually derived ROI. Histogram analysis was performed for assessment of tumor volume, tumor mass, density, skewness, kurtosis, and CT attenuation values at the 75th and 97.5th percentiles of Hounsfield units (HU). Texture parameters of uniformity, entropy, intensity variability, and size zone variability were also evaluated [36]. These CT parameters were evaluated on both non-contrast images and iodine contrast images.

Overall, the parameters analyzed include global parameters of solidity, size, volume, density, and mass of the tumor on CT, maximum standardized uptake value (SUVmax) on PET/CT, and all parameters obtained through histogram and texture analysis. We differentiated the CT parameters of the iodine map from those of non-contrast images by adding a lowercase “*i*” as a prefix. Gradient values of the CT parameters were also obtained, which represent the difference of the values obtained from non-contrast images and from the iodine map. These are indicated by the prefix “*g*”.

### Pathologic evaluation

**Figure 4:**
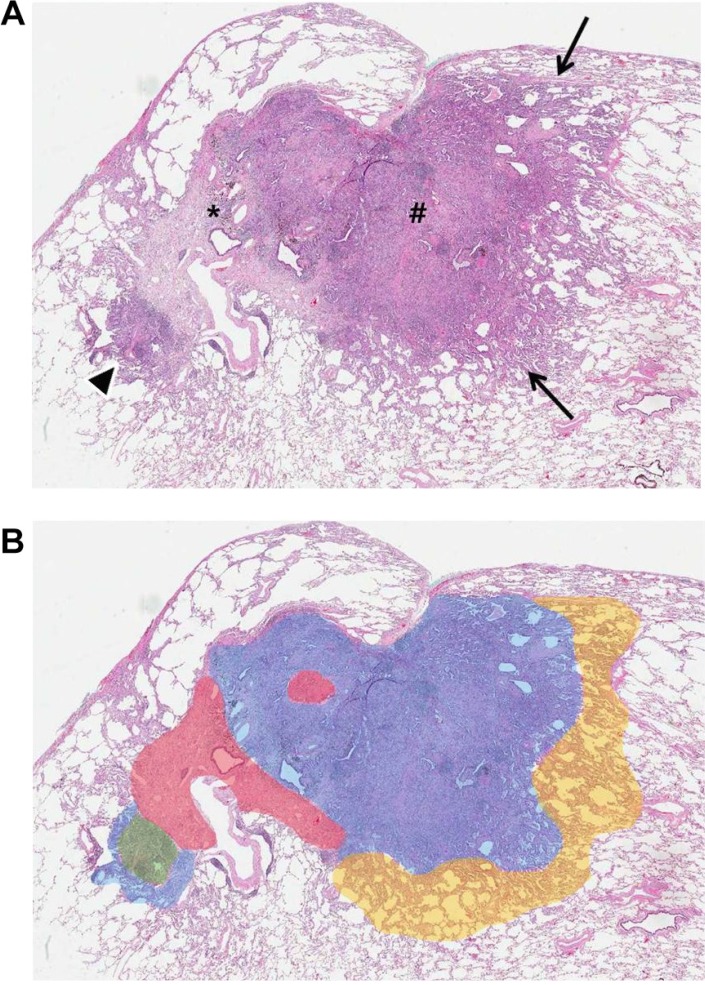
Lung adenocarcinoma in a 76-year-old woman (**A**) Photomicrograph shows internal scar tissue (*), surrounding areas of acinar and papillary (#) adenocarcinoma patterns, and lepidic pattern (arrows) (hematoxylin-eosin stain; original magnification, x10). (**B**) Schematic of tumor components shows estimated percentages of grade 1 (yellow area, 5%), grade 2 (blue area, 60%), grade 3 (green area, 5%), and central fibrosis (red area, 15%).

Each resected specimen (the entire tumor) was evaluated with standard pathologic methods as described in the surgical pathologic dissection manual of the Department of Pathology. All resected specimens were designated R0 (no residual tumor at the primary tumor site after surgical resection). For tumor sampling, an approximately 10-mm sample of tumor tissue from the entire tumor specimen was placed on a slide. All slides were scanned to produce a high-quality resolution digital image (0.25 lm/pixel at 40Å) using the Aperio Slide Scanning System (ScanScope T3; Aperio Technologies Inc., Vista, CA, USA). Two lung pathologists interpreted all tissue sections from virtual slides using ImageScope viewing software (Aperio Technologies, Inc.) and a high-resolution monitor [37]. For each case, the specimens were reviewed according to International Association for the Study of Lung Cancer (IASLC)/American Thoracic Society (ATS)/European Respiratory Society (ERS) International Multidisciplinary Lung Adenocarcinoma Classification criteria [38] and staged according to the seventh edition of the TNM classification for lung cancer. Comprehensive histologic subtyping was performed for the primary tumor in a semi-quantitative manner to the nearest 5% level, adding up to a total of 100% subtype components per tumor. The extent of the invasive component and tumor cellularity was measured and the most predominant subtype was recorded (Figure 4). When evaluating the predominant pattern, the central fibrosis area and its extent were disregarded. Next, the tumors were graded into 3 groups. Grade 1 included histologic subtypes of adenocarcinoma *in situ* (AIS), minimally invasive adenocarcinoma (MIA), and the lepidic pattern of invasive adenocarcinoma. Grade 2 corresponded to tumors that mainly showed acinar or papillary patterns and grade 3 corresponded to tumors that mainly showed micropapillary or solid patterns [21, 24, 26].

### Statistical analysis

Patient demographics and CT radiomics parameters were compared among the three different pathologic grades using one-way ANOVA with post hoc test of Bonferroni. A multinomial logistic regression model with the stepwise variable selection procedure using a 2-sided alpha of 5% as insertion and deletion criteria was used to predict three grades. In terms of variables for multivariate analysis, multicollinearity examination by using the variance inflation factor (VIF) was also performed. As for multiple nodules in a patient, we did not take into account within-patient correlation because each of them was considered as an independent synchronous lesion [39]. To evaluate the accuracy of the prediction of pathologic grade, we used leave-one-out cross-validation (CV) procedure and we constructed ROC curves plotting sensitivity versus 1-specificity, and calculated the area under the ROC curve (AUC), a measure of predictive power. Statistical analyses were performed using SAS version 9.4 (SAS Institute, Cary, NC, USA) and R 3.0.3 (Vienna, Austria; http://www.R-project.org). A *P value* less than 0.05 was considered to indicate a statistically significant difference.

The sample size calculation was based on the previous study by Sica et al. entitled “A Grading System of Lung Adenocarcinomas Based on Histologic Pattern is Predictive of Disease Recurrence in Stage I Tumors [24]. According to that report, the accuracy of finally selected grading system for concordance probability estimate was 0.65 (95% confidence interval 0.57–0.73) (p). The necessary sample size was calculated as follows: N = 4(Zcrit)2p(1- p)/D2, with a 90 % confidence interval (CI) of ± 10% (ie, Zcrit = 1.960) and where D = total width of the expected CI, that is, 0.20. Under these conditions, Power analysis indicated that a minimum sample size of 70 patients with lung adenocarcinoma.

## SUPPLEMENTARY MATERIALS



## Abbreviations

AIS: Adenocarcinoma in situ
AUC: Area under the receiver operating characteristic curve
CT: Computed tomography
DECT: Dual-energy computed tomography
FDG: Fluorodeoxyglucose
GGN: Ground-glass opacity nodule
GGO: Ground-glass opacity
HU: Hounsfield unit
MIA: Minimally invasive adenocarcinoma
NSCLC: Non-small cell lung cancer
PET: Positron emission tomography
ROC: Receiver operating characteristic
ROI: Region of interest
SUVmax: Maximum standardized uptake value
VNC: Virtual non-contrast
