# Supplementary material for: Pathologic stratification of operable lung adenocarcinoma using radiomics features extracted from dual energy CT images
Source: Oncotarget. 2016 Nov 21;8(1):523–35. doi: 10.18632/oncotarget.13476 (PMC5352175; doi:10.18632/oncotarget.13476)
Supplement: Supplementary file 1 [file oncotarget-08-523-s001.pdf]

Pathologic stratification of operable lung adenocarcinoma using radiomics features extracted from dual energy ct images

Supplementary Materials

Imaging and Analysis

All patients underwent CT examination using a dual-source CT system (Somatom Definition Flash; Siemens Healthcare, Forchheim, Germany) with the dual-energy technique. This DECT system was composed of two X-ray tubes and two corresponding 128-row detectors mounted in a perpendicular arrangement. DECT scanning was obtained 90 seconds after administration of contrast material (100 mL of iopamidol: Iomeron 300; Bracco, Milan, Italy) at a rate of 1.5 mL/sec using a power injector. This was followed by a 20-cc saline flush at a rate of 1.5 mL/sec. Imaging parameters were as follows: 105 mAs (effective) at 140 kV, 248 mAs (effective) at 80 kV, 32×0.6-mm collimation, pitch of 0.7, rotation time of 0.5 seconds, and a 512×512-pixel matrix. Imaging was performed from the thoracic inlet to the middle portion of the kidneys. Three different data sets were generated from the DECT imaging: 80 kV, 140 kV, and enhanced weighted average images. The weighted-average images were generated by combining the 140-kV and 80-kV data sets with a weighting factor of 0.6 (60% of information derived from the 80 kV image and 40% derived from the 140 kV image), thus these were approximately 120 kV images.

Data postprocessing and image reconstruction

The virtual non-enhanced images and iodine-enhanced images were generated using the liver Virtual Non-Contrast (VNC) application mode of dedicated dual-energy postprocessing software (Syngo Dual Energy; Siemens Medical Solutions). To obtain the iodine value of both solid and ground-glass opacity (GGO) components in each tumor, postprocessing was performed with two different software applications. For the solid component, the parameters for the material decomposition method were as follows: -110 HU for fat at 80 kV, -87 HU for fat at 140 kV, 52 HU for soft tissue at 80 kV, and 51 HU for soft tissue at 140 kV. For the GGO component, since the lesion was composed of a mixture of air and soft tissue the HU value of fat in the liver VNC application mode should be replaced with that of air, which is a HU value located at the interconnecting line between air and soft tissue. Thus, the material parameters were -110 HU for fat at 80 kV, -115 HU for fat at 140 kV, 60 HU for soft tissue at 80 kV, and 54 HU for soft tissue at 140 kV. Image data were reconstructed with a section thickness of 1 mm using a D30f (medium smooth) kernel for the iodine-enhanced image and a D45f (medium sharp) kernel for the virtual nonenhanced image.

RESULTS

Patient characteristics

All 101 consecutive patients underwent DECT and FDG-PET/CT for work-up. Among the 101 enrolled patients, 3 patients who were proven to have benign disease after percutaneous biopsy of the lesion and 5 patients who were proven to have unresectable stage III or stage IV lung cancer through further studies were excluded. One of the latter five patients was diagnosed as stage IV lung cancer due to brain metastasis detected on brain MRI, which was performed for staging work-up. Three of the five patients were diagnosed as stage III lung cancer due to lymph node metastasis, suggesting N2 or N3 disease that was detected through mediastinoscopic biopsy or endobronchial ultrasound-guided transbronchial needle aspiration (EBUS-TBNA). The remaining patient of the five was proven to have pleural metastasis during the operation, therefore no further resection was performed for the primary lung cancer. One additional patient who refused surgery was also excluded. Overall, 92 patients underwent complete resection including lobectomy or segmentectomy. Three patients with benign disease such as caseating granuloma were excluded postoperatively and eight patients who were diagnosed as stage III or IV due to pathologically proven lymph node metastasis or metastasis in a different ipsilateral lobe were additionally excluded. One patient with mucinous adenocarcinoma was also excluded because we included only patients with non-mucinous lung adenocarcinoma.

Supplementary Table S1: Correlation of pathologic stage with various pathologic features

Variables	Stage 1A (*n* = 72)	Stage 1B (*n* = 15)	Stage 2A (*n* = 3)	Stage 2B (*n* = 1)	*P*
Pathologic grade					
1: AIS/MIA/Lepidic	19	0	0	0	0.10
2: Acinar/Papillary	47	14	3	1	0.09
3: Micropapillary/Solid	6	1	0	0	0.96
Extent of invasion	12.9 ± 7.6	31.67 ± 11.8	36.67 ± 10.4	35.0 ± 0	**< 0.01***
Presence of invasion					
Vascular	0	1	0	0	0.17
Lymphatic	11	4	0	1	0.09
Perineural	0	1	0	1	**< 0.01***
Pleural	2	2	2	1	**< 0.01***
Tumor cellularity (%)	49.1 ± 14.3	51.4 ± 16.1	46.7 ± 15.3	60.0 ± 0	0.82

*$P < 0.05$
*P values were calculated with one-way ANOVA.
